# Supplementary material for: Cardiorespiratory fitness in late adolescence and long-term risk of psoriasis and psoriatic arthritis among Swedish men
Source: PLoS One. 2021 Jan 11;16(1):e0243348. doi: 10.1371/journal.pone.0243348 (PMC7799831; doi:10.1371/journal.pone.0243348)
Supplement: S1 Table — Baseline data and age at first psoriasis and psoriatic arthritis diagnosis in 582,937 male conscripts with CRF data missing. For detailed information on parental education levels, please see “Register data and classification of psoriasis and psoriatic arthritis”. Unless otherwise specified, data are presented as means ± standard deviation (SD) or n (%). IQR = interquartile range. (DOCX) [file pone.0243348.s001.docx]

**S1 Table. Baseline data on conscripts with missing data on cardiorespiratory fitness (CRF).**

| **Parameters** | | **All**  (N=582,937) | **Median age (years) at diagnosis (IQR)** | |  |  |
| --- | --- | --- | --- | --- | --- | --- |
| **Age (years)** | | 18.4±0.81 |  | |  |  |
| **Height (m)** | | 1.80±0.07 |  | |  |  |
| **Weight (kg)** | | 71.8±12.9 |  | |  |  |
| **Body mass index (kg/m^2^)** | | 22.3±3.67 |  | |  |  |
| **Parental education (highest achieved level)** | |  |  | |  |  |
| **1–2** | | 115,911 (20.5%) |  | |  |  |
| **3–4** | | 261,859 (46.3%) |  | |  |  |
| **5–7** | | 187,674 (33.2%) |  | |  |  |
| **Psoriasis and/or psoriatic arthritis**  **Psoriasis**  **Psoriatic arthritis** | | 9,531 (1.6%)  8,598 (1.5%)  2,156 (0.4%) | 35.0 (29.0–44.0)  34.5 (28.0–43.0)  38.0 (31.0–47.0) | |  |  |
|  |  |  |  |  | |  |
