# Supplementary material for: Cardiorespiratory fitness in late adolescence and long-term risk of psoriasis and psoriatic arthritis among Swedish men
Source: PLoS One. 2021 Jan 11;16(1):e0243348. doi: 10.1371/journal.pone.0243348 (PMC7799831; doi:10.1371/journal.pone.0243348)
Supplement: S2 Table — Data are presented as crude incidence rates (95% confidence interval (CI)). PYs = person-years. S2A Table represents incidence rates for 1968–2000, and S2B Table for 2001–2016. (DOCX) [file pone.0243348.s002.docx]

**S2 Table. Incidence of psoriasis and psoriatic arthritis in conscripts with missing data on cardiorespiratory fitness (CRF)** (n=582,937)**.**

**A**

| **Diagnosis** |  |
| --- | --- |
| **Psoriasis and/or psoriatic arthritis**  N  Cases per 100,000 PYs | 271  5.7  (5.1–6.4) |
| **Psoriasis**  N  Cases per 100,000 PYs | 211  4.4  (3.9–5.1) |
| **Psoriatic arthritis**  N  Cases per 100,000 PYs | 76  1.6  (1.3–2.0) |

**B**

| **Diagnosis** |  |
| --- | --- |
| **Psoriasis and psoriatic arthritis**  N  Cases per 100,000 PYs | 9,260  110.5  (108.3–112.8) |
| **Psoriasis**  N  Cases per 100,000 PYs | 8,387  100.0  (97.9–102.2) |
| **Psoriatic arthritis**  N  Cases per 100,000 PYs | 2,080  24.7  (23.6–25.8) |
