# Supplementary material for: Cardiorespiratory fitness in late adolescence and long-term risk of psoriasis and psoriatic arthritis among Swedish men
Source: PLoS One. 2021 Jan 11;16(1):e0243348. doi: 10.1371/journal.pone.0243348 (PMC7799831; doi:10.1371/journal.pone.0243348)
Supplement: S3 Table — Hazard ratios (95% confidence intervals (CIs)) for psoriasis and/or psoriatic arthritis with respect to cardiorespiratory fitness (CRF) among those conscripted after 1995. (DOCX) [file pone.0243348.s003.docx]

**S3 Table. Hazard ratios (HRs) for psoriasis and/or psoriatic arthritis in men conscripted after 1995.**

| **Diagnosis** | | | **Number**  (events/population) | | **HR (95% CI) per cardiorespiratory fitness category** | | | | |
| --- | --- | --- | --- | --- | --- | --- | --- | --- | --- |
|  | |  | | | **High**  (7**–**9)  *Reference* | **Medium**  (4**–**6) | | **Low**  (1**–**3) | |
| **Psoriasis and/or psoriatic arthritis**  Model 1  Model 2  Model 3  Model 4 | | | 1,536/154,221  1,536/154,221  1,535/154,215  1,508/152,229 | | 1.00  1.00  1.00  1.00 | 1.16 (1.05–1.29)  1.16 (1.05–1.29)  1.15 (1.03–1.28)  1.13 (1.02–1.26) | | 1.80 (1.18–2.74)  1.80 (1.18–2.74)  1.46 (0.92–2.30)  1.34 (0.83–2.16) | |
|  |  | | |  | |  |  | |  |
|  |  | | |  | |  |  | |  |

Model 1: Adjusting for age at conscription, year of conscription, and conscription test centre.

Model 2: Additionally adjusting for alcohol abuse and diabetes mellitus at conscription.

Model 3: Additionally adjusting for body mass index at conscription.

Model 4: Additionally adjusting for parental education.
